# NF-κB-Associated Pain-Related Neuropeptide Expression in Patients with Degenerative Disc Disease

**DOI:** 10.3390/ijms20030658

**Published:** 2019-02-03

**Authors:** Aisha S. Ahmed, Svante Berg, Kanar Alkass, Henrik Druid, David A. Hart, Camilla I. Svensson, Eva Kosek

**Affiliations:** 1 Department of Clinical Neuroscience, Karolinska Institutet, 171 77 Stockholm, Sweden; Eva.Kosek@ki.se; 2 Stockhom Spine Center, Löwenströmska Hospital, 194 89 Upplands Väsby, Sweden; svante.berg@spinecenter.se; 3 Department of Oncology-Pathology, Karolinska Institutet, 171 77 Stockholm, Sweden; kanar.alkass@ki.se (K.A.); henrik.druid@ki.se (H.D.); 4 Swedish National Board of Forensic Medicine, 171 65 Solna, Sweden; 5 McCaig Institute for Bone & Joint Health, University of Calgary, Calgary, AB T2N 1N4, Canada; hartd@ucalgary.ca; 6 Department of Physiology and Pharmacology, Karolinska Institutet, 17177 Stockholm, Sweden; Camilla.Svensson@ki.se

**Keywords:** nuclear factor-κB (NF-κB), chronic low back pain, degenerative disc disease (DDD), calcitonin gene related peptide (CGRP), substance P (SP), transient receptor potential V (TRPV)

## Abstract

The role of nuclear factor kappa-light-chain-enhancer of activated B cells (NF-κB) has been highlighted in mechanisms underlying inflammatory and neuropathic pain processes. The present study was designed to investigate whether NF-κB signaling is associated with pain-related neuropeptide expression in patients with chronic back pain related to degenerative disc disease (DDD). Intervertebral disc (IVD) tissues were collected from forty DDD patients undergoing disc replacement or fusion surgery, and from eighteen postmortem (PM) control subjects. *RELA*, *NFKB1*, *CGRP*, *TAC1*, *TRPV1*, and *MMP-3* gene expression were analyzed by RT-qPCR, while NF-κB subunit RelA and NF-κB1–DNA binding in nuclear extracts and calcitonin gene related peptide (CGRP), substance P (SP), and transient receptor potential, subfamily V, member 1 (TRPV1) protein levels in cytosolic extracts of tissues were assessed by enzyme-linked immunosorbent assay (ELISA). An upregulated NF-κB1–DNA binding, and higher CGRP and TRPV1 protein levels were observed in DDD patients compared to PM controls. In DDD patients, NF-κB1–DNA binding was positively correlated with nuclear RelA levels. Moreover, NF-κB1–DNA binding was positively associated with *TRPV1* and *MMP-3* gene and SP and TRPV1 protein expression in DDD patients. Our results indicate that the expression of SP and TRPV1 in IVD tissues was associated with NF-κB activation. Moreover, NF-κB may be involved in the generation or maintenance of peripheral pain mechanisms by the regulation of pain-related neuropeptide expression in DDD patients.

## 1. Introduction

Degenerative disc disease (DDD) is an important cause of chronic low back pain [1]. DDD is characterized by the loss of extracellular matrix, initiated by an imbalance between catabolic and anabolic mediator expression in the chondrocytes and in matrix [2]. Under normal conditions, intervertebral discs (IVDs) are sparsely innervated and the innervation is limited to the outer annulus fibrosus (AF) [3]. In patients suffering from DDD, sensory and autonomic fibers are reported at the outer AF aspects of the IVD, as well as at the inner nucleus pulposus (NP) [4,5], indicating that nerve ingrowth along fissures in the disc are potentially associated with algogenic mechanisms in DDD. 

An upregulated expression of calcitonin gene related peptide (CGRP) and substance P (SP) along with nerve growth factor (NGF) in sensory nerves innervating IVD has been reported in DDD patients [6,7]. Additionally, higher levels of pro-inflammatory cytokines; tumor necrosis factor-alpha (TNF-α), interleukin (IL)-6 and IL-8 and matrix metalloproteinase (MMP)-3, MMP-10, MMP-13 have been observed in painful degenerative discs [7,8]. Further, it has been postulated that persistent disc inflammation stimulates the axonal regeneration of sensory nerve fibers [5,9], suggesting that inflammatory processes may play a significant role in chronic back pain. Nuclear factor kappa-light-chain-enhancer of activated B cells (NF-κB) is one of numerous signaling pathways reported to play a critical role in inflammatory degenerative processes [10,11]. NF-κB-mediated inflammatory responses in chondrocytes can lead to progressive extracellular matrix damage [12]. In addition, various pre-clinical studies have reported on the role of NF-κB and its downstream pro-inflammatory cytokines in mechanisms underlying inflammatory and neuropathic pain processes [13,14]. NF-κB is a family of transcription factors mainly consist of two subunits; RelA/p65 and NF-κB1/p50. The mammalian NF-κB sub-family comprises five proteins: RelA/p65, RelB, c-Rel, NF-κB1/p50 and NF-κB2/p52. In different cell types NF-κB can be present as homo- or heterodimers [15,16]. Canonical NF-κB activation pathway involves activation of predominantly RelA/NF-κB1 heterodimers [16]. Under normal conditions, NF-κB is retained in the cytoplasm. Pro-inflammatory cytokines, excessive mechanical stress or matrix degradation can initiate a cascade of reactions leading to the IκB kinase (IKK) dependent NF-κB activation and subsequent translocation of NF-κB to the nucleus that can induce gene transcription [15]. Activation of NF-κB, as measured by the upregulated expression of RelA, has been reported in IVD tissues extracted from patients with DDD [17]. By blocking nuclear RelA translocation with specific inhibitors, it has been shown that the process of IVD degeneration was decreased in a rat model of DDD [18], suggesting a role for NF-κB in IVD degenerative processes. Further, the intra-discal inhibition of IKKβ has been shown to significantly downregulate CGRP expression in the cell bodies of dorsal root ganglia (DRG) neurons innervating rat IVD [19], demonstrating that NF-κB activation in residual cells has an impact on sensory neurons innervating IVD tissues. The systemic inhibition of NF-κB has been shown to downregulate the CGRP and SP expression in inflamed joints and in corresponding DRG and reversed pain behavior in animal models of arthritis [20,21]. However, the role of NF-κB signaling on SP expression or NF-κB-related processes in human IVD tissues likely influencing nociceptive signaling in DDD patients have, to our knowledge, not been studied before. 

The transient receptor potential, subfamily V, member 1 (TRPV1) is an ion channel present at the central and peripheral nervous system and is highly expressed in CGRP and SP positive afferent sensory neurons [22]. Preclinical studies have provided evidence for the contribution of TRPV1 to chronic pain processes [23,24]. TRPV1 has been reported to play a role in the activation of the NF-κB signaling in microglia cells cultured from rat retina [25]. However, TRPV1 expression in human IVD tissues or its potential role in mechanisms underlying chronic back pain has not been reported previously.

Focusing on the peripheral pain mechanisms in patients with DDD, we aimed to investigate the relationship between NF-κB signaling and the expression of sensory neuropeptides CGRP and SP in IVD tissues and to study whether this relationship involves TRPV1 expression regarding pain severity. For this purpose, we have quantified NF-κB1–DNA binding and nuclear RelA levels, as well as studied the expression of CGRP, SP, and TRPV1 both at the mRNA and protein levels in IVD tissues collected from DDD patients and postmortem (PM) controls. As L4-S1 segments of spine are the most common source of back pain in humans [26], we chose to analyze IVD tissues collected from these segments. As a marker of degeneration, we further measured the gene expression of matrix metalloprotease-3 (MMP-3), one of many proteases involved in disc degeneration and regulated by NF-κB signaling [16,27]. Considering that inflammatory processes are reported to initiate a cascade of reactions leading to IVD degeneration and causing back pain through the release of algogenic neuropeptides, we hypothesized that NF-κB (RelA/NF-κB1) activation and the regulation of CGRP, SP, and TRPV1 expression would correlate positively in IVD tissues collected from patients with DDD. In an exploratory part of the study, we also assessed potential associations of CGRP, SP, and TRPV1 tissue expression with symptom severity.

## 2. Results

### 2.1. Patients’ Demographic and Clinical Data 

The studies included forty patients with DDD (average age 44.5 ± 10.0), and eighteen PM controls (average age 42.7 ± 13.0 years) with no statistically significant age differences between the groups (*p* = 0.56). Body mass index (BMI) was significantly lower (*p* = 0.0001) in patients compared to PM controls and recorded as 25.4 ± 5.4 for patients and 28.37 ± 5.1 for PM controls. All patients reported pain intensities for the back and legs and the reported scores were 32.01 (±19.98) and 4.71 (±9.37), respectively, indicating moderate back pain and low intensities of referred leg pain in these patients. The DDD patients reported average ODI scores of 31.59 (±12.25) indicating moderate disability (Table 1).

### 2.2. NF-κB Expression and Activity

**Figure 1:**
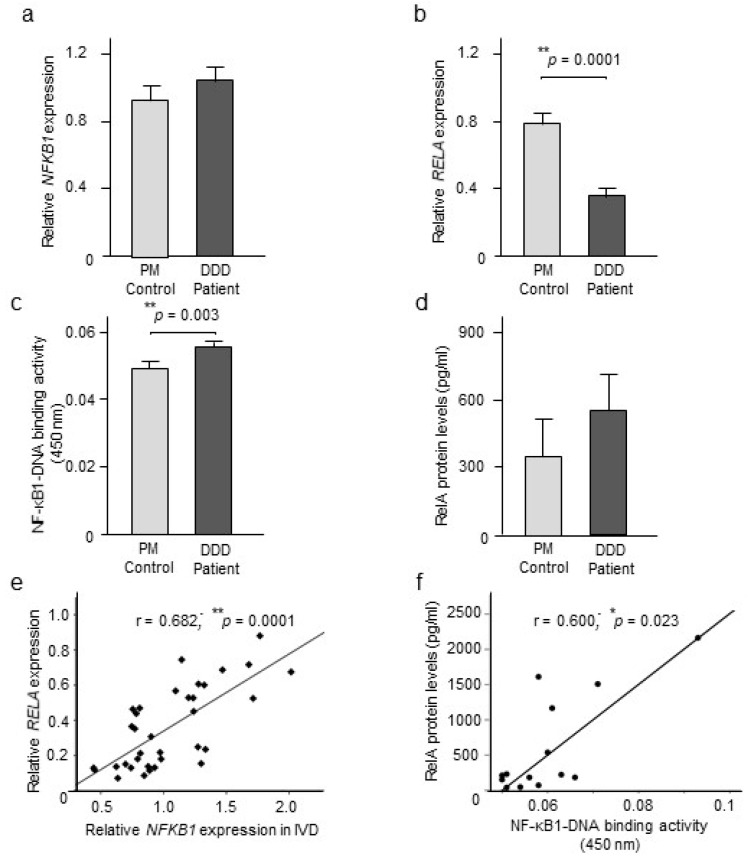
NF-κB expression and activation state in intervertebral disc (IVD) tissues. (**a**) Relative expression of *NFKB1* (*n* = 33 DDD patients and 17 PM control samples) and *(***b**) *RELA* gene expression (*n* = 33 DDD patients and 16 PM control samples) in the IVD tissues retrieved from DDD patients and PM controls. Values reported are mean ± SEM. ** *p* ≤ 0.01 compared to PM control calculated by Student’s *t*-test. (**c**) Nuclear NF-κB1–DNA binding activity in IVD extracted from DDD patients and PM controls. Values reported are mean ± SEM. *n* = 34 for DDD patients and 18 for PM controls. ** *p* ≤ 0.01 compared to PM control calculated by Student’s *t*-test; (**d**) Nuclear RelA levels in IVD extracted from DDD patients and PM controls. Values reported are mean ± SEM. *n* = 15 for DDD patients and 3 for PM controls. (**e**) Correlation among *NFKB1* mRNA levels and *RELA* gene expression in DDD patients (*r* = 0.682; *p* = 0.0001; *n* = 35; Spearman correlation co-efficient), and (**f**) Correlation among nuclear NF-κB1–DNA binding activity and RelA levels in IVD from DDD patients (*r* = 0.600; *p* = 0.023; *n* = 14; Spearman correlation co-efficient).

Our quantitative RT-PCR analysis showed no statistically significant differences in *NFKB1* mRNA levels in IVD tissue collected from DDD patients and the PM controls (Figure 1a), whereas a downregulation (*p* = 0.0001) of *RELA* mRNA levels was observed for DDD patients compared to the PM controls (Figure 1b). 

Analysis of nuclear extracts revealed that NF-κB1–DNA binding activity was significantly (*p* = 0.003) upregulated in DDD patients compared to the PM controls (Figure 1c). Moreover, a trend for higher RelA levels, although not significant most likely due to limited number (*n* = 3) of subjects positive for the signal in PM group, was observed for the DDD patients compared to PM controls (Figure 1d), potentially indicating increased nuclear NF-κB translocation in the disease state. No age, gender or BMI related changes were detected for NF-κB1–DNA binding activity in DDD patients and PM controls as assessed by univariate analyses of covariance. 

To measure NF-κB activation, we assessed associations between the two NF-κB subunits at the mRNA and protein levels in IVD tissues. A positive correlation (*r* = 0.682; *p* = 0.0001; *n* = 35) was observed between *NFKB1* and *RELA* mRNA levels in DDD patients (Figure 1e). Similarly, a positive association (*r* = 0.600; *p* = 0.023; *n* = 14) was observed between nuclear NF-κB1–DNA binding and nuclear RelA levels in DDD patients (Figure 1f). No effects of age, gender, or BMI on outcome of association among *NFKB1* and *RELA* gene expression or between nuclear RelA and NF-κB1–DNA binding activity was observed in DDD patients as examined by partial correlation analysis. 

### 2.3. MMP-3 Gene Expression and Association with NF-κB Signaling 

**Figure 2:**
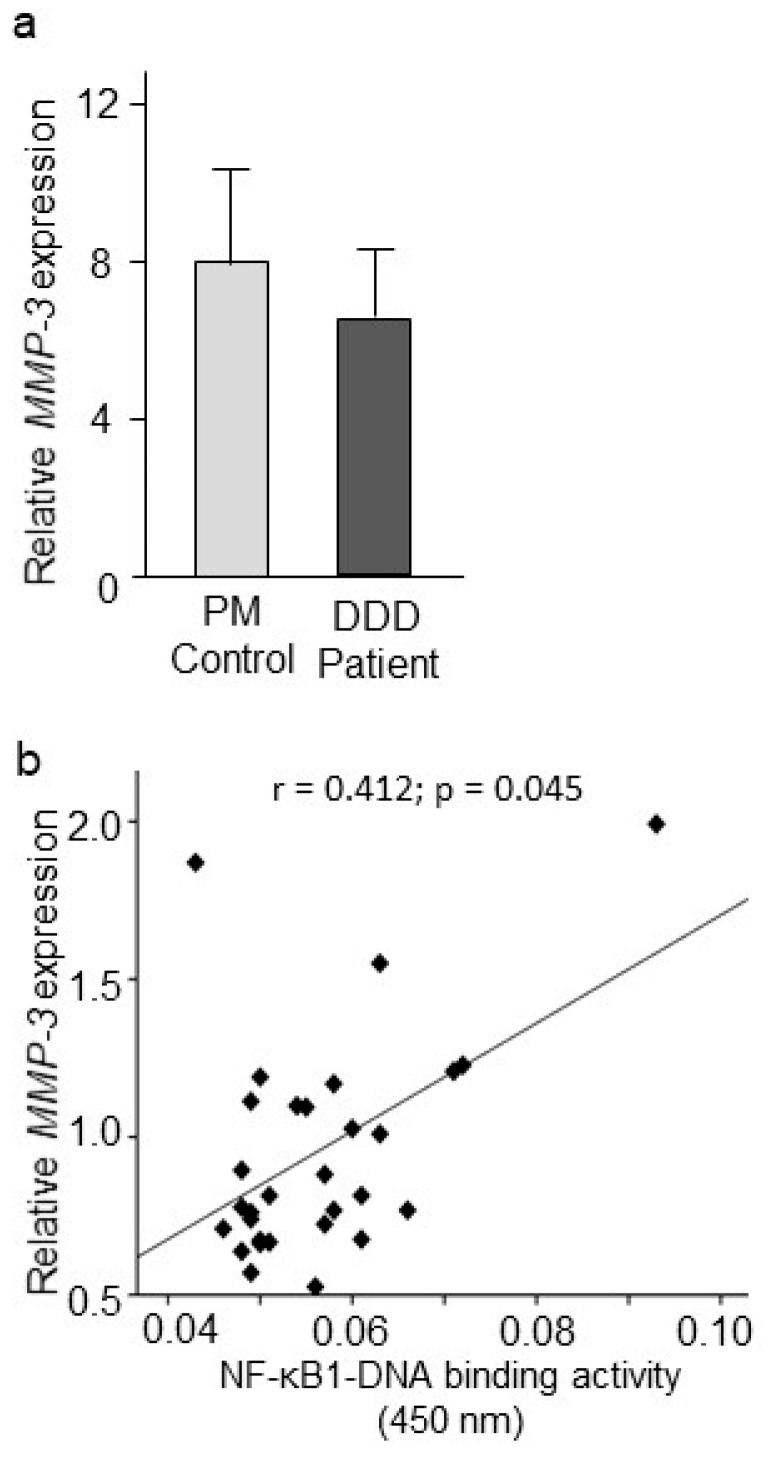
*MMP-3* gene expression and association to NF-κB. (**a**) Relative expression of *MMP-3* in the IVD tissues retrieved from DDD patients and PM controls. Values reported are mean ± SEM. *n* = 33 DDD patients and 17 PM control samples; and (**b**) Correlation between nuclear NF-κB1–DNA binding and *MMP-3* gene expression (*r* = 0.412; *p* = 0.045; *n* = 29; Spearman correlation co-efficient).

Our quantitative RT-PCR analysis did not detect any statistically significant differences in *MMP-3* mRNA levels in IVD tissue collected from DDD patients and the PM controls (Figure 2a). However, a positive correlation was observed between *MMP-3* gene expression and NF-κB1–DNA binding activity (*r* = 0.412; *p* = 0.045; *n* = 29) when corrected for age, gender, and BMI effects within the patient group (Figure 2b).

### 2.4. CGRP, SP, and TRPV1 Expression

**Figure 3:**
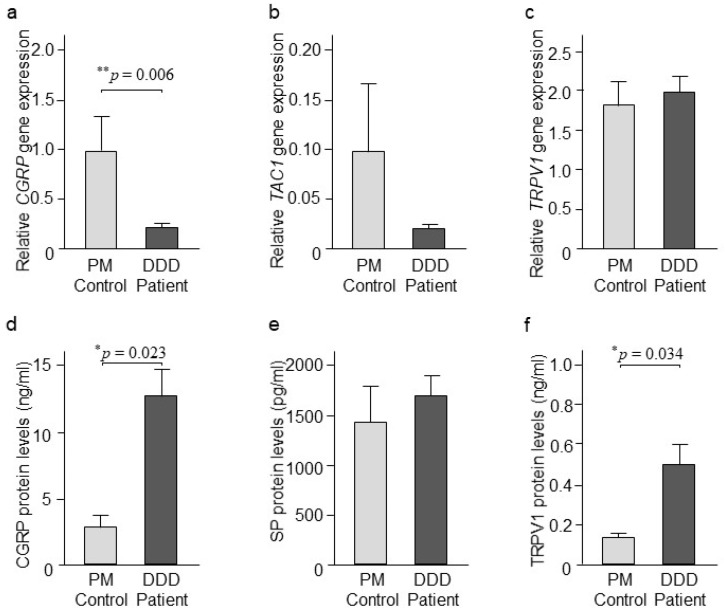
CGRP, SP, and TRPV1 expression. (**a**) Relative gene expression of *CGRP* (*n* = 23 DDD patients and 14 PM control), (**b**) *TAC1* (*n* = 33 DDD patients and 16 PM control), and (**c**) *TRPV1* (*n* = 33 DDD patients and 17 PM control) in intervertebral disc (IVD) tissues retrieved from DDD patients and PM controls. Values reported are mean ± SEM. ** *p* ≤ 0.01, comparison between DDD patients and PM controls calculated by Student’s *t*-test. (**d**) Protein levels of CGRP (*n* = 30 DDD patients and 7 PM control), (**e**) SP (n = 25 DDD patients and 9 PM control), and (**f**) TRPV1 (*n* = 28 DDD patients and 9 PM control) in cytosolic extracts retrieved from IVD tissues from DDD patients and PM controls. Values reported are mean ± SEM. * *p* ≤ 0.05, comparison between DDD patients and PM controls calculated by Student’s *t*-test.

Quantitative RT-PCR analysis showed significantly downregulated (*p* = 0.006) *CGRP* mRNA levels in DDD patients compared to the PM controls. No changes were detected for *Tachykinin precursor 1* (*TAC1*), gene which encode for SP, or *TRPV1* mRNA levels between DDD patients and the PM controls (Figure 3a–c). No effects of age, gender, or BMI were detected for *CGRP* gene expression between DDD patients and PM controls as measured by univariate analyses of covariance. Our ELISA analysis revealed upregulated (*p* = 0.023) CGRP and TRPV1 (*p* = 0.034) expression in DDD patients compared to PM controls. No changes were detected for SP expression in tissue from DDD patients compared to the PM controls (Figure 3d–f). No effects of age, gender, or BMI were detected for differences between DDD patients and PM controls for CGRP and TRPV1 levels except that of BMI for TRPV1 as measured by univariate analyses of covariance. 

### 2.5. Association among CGRP, SP, and TRPV1 

**Figure 4:**
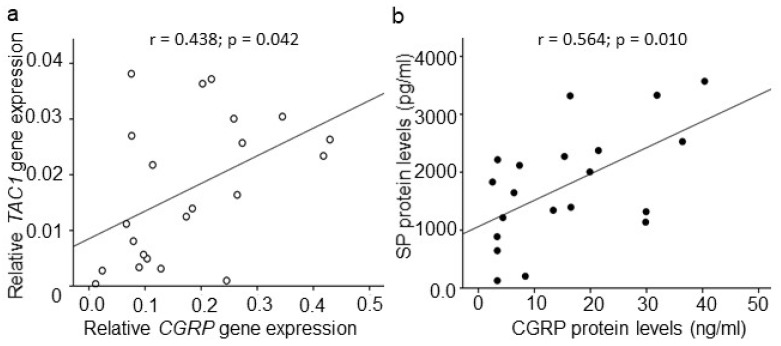
Correlations among CGRP and SP expression in DDD patients. (**a**) Correlation among *TAC1* and *CGRP* gene expression (*r* = 0.438; *p* = 0.042; *n* = 22; Spearman correlation co-efficient) and, (**b**) Correlation among CGRP and SP (*r* = 0.564; *p* = 0.010; *n* = 20; Spearman correlation co-efficient) in IVD tissues.

A positive correlation was observed between *CGRP* and *TAC1* gene expression (*r* = 0.438; *p* = 0.042; *n* = 22) in IVD tissues collected from DDD patients (Figure 4a). Moreover, CGRP and SP protein levels were found to be positively correlated (*r* = 0.564; *p* = 0.010; *n* = 20) in the patient group (Figure 4b) no age, gender, or BMI effects were seen. No statistically significant correlations were observed between *CGRP* and *TRPV1* gene expression, or between *TAC1* and *TRPV1* mRNA levels in DDD patients. In addition, no correlations were observed between CGRP and TRPV1, however, a trend towards significance (*r* = 0.454; *p* = 0.051; *n* = 19) was observed between SP and TRPV1 protein levels in DDD patients.

### 2.6. Association between CGRP, SP, TRPV1, and NF-κB Signaling

**Figure 5:**
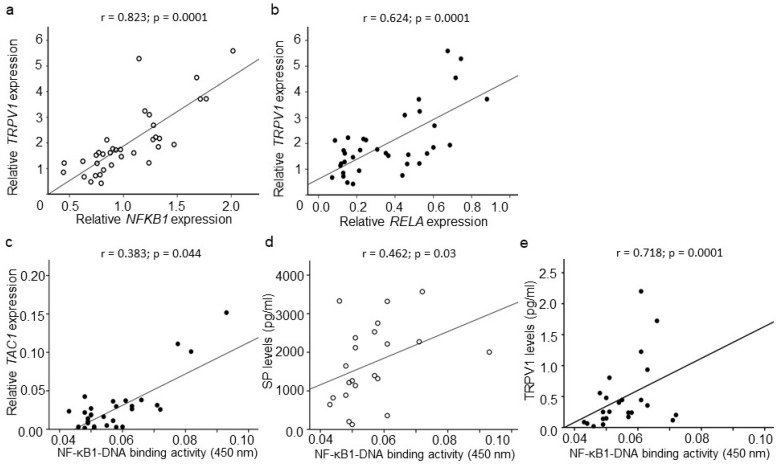
Relationship among NF-κB, SP and TRPV1 expression in DDD patients. Correlations between (**a**) *NFKB1* and *TRPV1* gene expression (*r* = 0.823; *p* = 0.0001; *n* = 35), and (**b**) *RELA* and *TRPV1* gene expression (*r* = 0.624; *p* = 0.0001; *n* = 35). Correlations between nuclear NF-κB1–DNA binding and (**c**) *TAC1* gene expression (*r* = 0.383; *p* = 0.044; *n* = 28), (**d**) SP (*r* = 0.462; *p* = 0.03; *n* = 22), and (**e**) TRPV1 (*r* = 0.718; *p* = 0.0001; *n* = 24) protein levels in IVD tissues collected from DDD patients, All associations were made by Spearman correlation co-efficient.

A positive correlation was observed between *NFKB1* and *TRPV1* gene expression (*r* = 0.823; *p* = 0.0001; *n* = 35) and between *RELA* and *TRPV1* mRNA (*r* = 0.624; *p* = 0.0001; *n* = 35) levels in IVD tissues collected from DDD patients (Figure 5a,b). No correlations were observed between *NFKB1* and *CGRP* or *TAC1* gene expression, or between *RELA* and *CGRP* or *TAC1* gene expression for the DDD patients. 

Nuclear NF-κB1–DNA binding correlated positively with *TAC1* mRNA levels (*r* = 0.383; *p* = 0.044; *n* = 28) in DDD patients (Figure 5c). No correlations were observed between NF-κB1–DNA binding and *CGRP* or *TRPV1* gene expression, and between nuclear ReLA levels and each of *CGRP*, *TAC1,* or *TRPV1* gene expression in DDD patients. However, nuclear NF-κB1–DNA binding correlated positively with SP (*r* = 0.462; *p* = 0.03; *n* = 22) and with TRPV1 (*r* = 0.718; *p* = 0.0001; *n* = 24) protein levels in IVD tissues extracted from DDD patients (Figure 5d,e). No correlations were observed between NF-κB1–DNA binding and CGRP or between nuclear ReLA levels and CGRP, SP, or TRPV1 expression in DDD patients. No effects of age, gender, or BMI were obvious for any of the associations observed. 

### 2.7. Association between NF-κB, CGRP, SP, TRPV1, and Clinical Symptoms

In DDD patients, *TAC1* gene expression correlated negatively with back pain (*r* = −0.397; *p* = 0.027; *n* = 31). No associations were observed between pain at the spine with any of the *RELA*, *NFKB1*, *CGRP,* or *TRPV1* gene expression. A positive correlation was observed between ODI and *TRPV1* gene expression (*r* = 0.377; *p* = 0.028; *n* = 34) and a trend towards significance was seen between ODI and *NFKB1* mRNA levels (*r* = 0.305; *p* = 0.079; *n* = 34). No association were obvious among ODI and *RELA*, *CGRP* or *TAC1* gene expression in DDD patients. No associations were observed for any of the clinical parameter with NF-κB1–DNA binding or with CGRP, SP, or TRPV1 protein expression except that of nuclear ReLA levels which correlated negatively with ODI (*r* = −0.507; *p* = 0.045; *n* = 16) in DDD patients. 

## 3. Discussion

The main findings of the present studies are that NF-κB1–DNA binding and CGRP and TRPV1 levels were higher in IVD tissues extracted from DDD patients compared to PM controls. The upregulated NF-κB1–DNA binding correlated positively with nuclear RelA levels, as well as with cytosolic SP and TRPV1 protein levels in these patients. Positive correlations were also observed between CGRP and SP, both at the mRNA and protein levels in IVD tissues collected from DDD patients. Thus, these results suggest that the pathological activation of the NF-κB signaling is involved in intervertebral disc degeneration and may modulate peripheral pain processes underlying chronic back pain. The study also identified the presence of TRPV1 within IVD tissue, and determined its association with the NF-κB activation. 

Pathological NF-κB activation has been reported for numerous degenerative diseases, such as osteoarthritis [28], rheumatoid arthritis [11], and muscular dystrophy [29]. Here, we observed upregulated NF-κB1–DNA binding in IVD tissues collected from DDD patients, indicating NF-κB1 translocation to the nucleus, an essential step in NF-κB activation [15]. Although, no changes were detected at nuclear RelA levels in DDD patients in comparison to PM control group, most likely due to limited number of subjects in the control group, RelA levels positively correlated with NF-κB1–DNA binding indicating NF-κB (RelA/NF-κB1) activation in DDD patients. Activation of NF-κB signaling in human IVD tissues has been reported previously, particularly in the NP [18,19] where NF-κB activation measured as higher phosphorylated-RelA levels was reported in IL-1β stimulated human NP cells [19]. Further, NF-κB activity was shown to be related to the oxidative stress and degree of IVD degeneration [30]. In animal models of disc degeneration, intra-discal injections of NF-κB decoy oligonucleotides restored IVD height [31], indicating that activation of NF-κB is involved in matrix degradation. Our present findings strengthen previous observations of NF-κB activation in degenerative IVD tissues, but also provide evidence of activated NF-κB signaling in IVD NF tissues in DDD patients. 

In human IVD tissues, we observed an association of NF-κB1–DNA binding with SP expression, as well with *TAC1* gene expression in DDD patients. Our findings raise the possibility that NF-κB may regulate pain-related mediator expression in IVD tissues as well in sensory neurons innervating IVD tissues. This relationship can be a critical link in the cycle of pain and inflammation that perpetuates in chronic painful conditions. The role of NF-κB and its downstream pro-inflammatory mediators in mechanisms underlying inflammatory and neuropathic pain processes has been highlighted [14,32]. In a nerve-injury model, the administration of NF-κB decoys attenuated thermal hyperalgesia [33]. The intrathecal administration of a NF-κB inhibitor has been shown to reverse the mechanical allodynia in neuropathic pain in a rat model [34]. Our previous work has shown that systemic NF-κB inhibition can reverse inflammatory pain and reduce SP and CGRP expression in joints, and in the dorsal root ganglion (DRG) in animal models of arthritis [20,32]. Interestingly, the intra-discal inhibition of IKKβ has been shown to significantly downregulate CGRP expression in the cell bodies of DRG neurons innervating the rat IVD [19]. Our present findings endorse previous observations of NF-κB-related CGRP expression in disc tissues but also provide evidence of NF-κB associated expression of SP in human IVD tissues. In contrast, the regulatory functions of SP and CGRP on the NF-κB signaling has also been highlighted [35,36]. CGRP has been reported to regulate NF-κB activation by interfering with phosphorylation and degradation of the IκB in mouse thymocytes [35]. While, SP has been demonstrated to increase the secretion of cytokines via the activation of NF-κB and mitogen-activated protein kinases (MAPK), two of the most important signaling pathways in IVD [36]. Thus, demonstrating the regulation of the NF-κB signaling by pain mediating peptides. However, further studies are needed to clarify the interactions between NF-κB and SP and CGRP in order to delineate the underlying mechanisms related to chronic back pain associated with intervertebral disc degeneration. 

The release of SP from sensory nerves or from disc cells into the matrix can potentially upregulate the production of matrix-degrading enzymes and also sensitize ingrowing nerves resulting in nociceptive transmission, one of the mechanisms involved in chronic back pain. Indeed, it has been shown that SP can increase collagen remodeling by enhancing MMP-3 expression [37]. In the present study, we were unable to observe the upregulated expression of SP in IVD tissues collected from DDD which might have had an effect on MMP-3 expression as no changes were detected for *MMP-3* mRNA expression in DDD compared to PM control tissues. Higher MMP-3 and MMP-9 levels are reported in degenerative compared to less-degenerative discs [38] and that NF-κB inhibition downregulated the TNFα and MMP-3 and -9 expression in IVD tissues [16,17]. In the current study, while no changes were detected for *MMP-3* mRNA expression, *MMP-3* gene expression was associated with NF-κB1–DNA binding confirming the strong potential for NF-κB-mediated transcription of inflammatory and degenerative genes in IVD tissues, leading to matrix degradation. 

We observed higher CGRP protein levels while for SP, no statistically significant differences were observed at the protein or mRNA levels in IVD tissues retrieved from DDD patients. Interestingly, increased CGRP levels correlated positively with SP expression both at the mRNA and protein levels in disc tissues indicating their co-expression and synthesis by degenerative disc cells as well in nerve terminal innervating IVD tissues in DDD patients. An upregulated CGRP and SP expression has been reported in sensory nerves innervating degenerated IVD in DDD patients [5,6,7]. Furthermore, an increased SP expression has been reported in TNF-α stimulated human NP cells and in NP tissues extracted from elderly female DDD patients [39,40]. However, we could not observe higher SP levels in IVD tissues collected from DDD patients in comparison to PM controls. For the current study, changes in cellular permeability in deceased may have modified levels of SP in PM tissues. Indeed, higher SP levels are detected in PM brain tissue and upregulated serum SP levels are reported to be associated with patient’s mortality after brain injuries [41,42]. 

A role of CGRP and SP in pain has been widely reported in chronic painful conditions [19,43]. In a rat model of disc inflammation, CGRP-positive nerve fibers were reported in inflamed disc [44] indicating CGRP as a potential inflammatory pain-related marker in DRG-innervating animal IVD. However, we could not find any associations between clinical symptoms and either of CGRP or SP expression in DDD patients. One of the probable causes behind differences between the results of previous studies and ours may reflect the fact that our results are based on combined neuronal and cellular levels while previous studies focus mainly on the neuronal source of CGRP and SP expression. Further, the pre-medication before surgery could have affected the CGRP or SP expression or might have influenced the peripheral or central mechanisms of pain in DDD patients. 

In tissues collected from DDD patients and PM controls, we were able to measure *TRPV1* gene expression, suggesting its synthesis both under normal and pathological conditions in IVD tissues. Non-neuronal TRPV1 synthesis has been reported for chondrocytes, osteoclasts, and synovial fibroblasts [45,46,47] and the presence of TRPV1 in sensory neurons innervating bone in an osteoporotic animal model [48] indicates that TRPV1 is not limited to pain sensation, but it may also participate in degeneration processes. Despite no changes detected at the mRNA level, higher TRPV1 protein levels were seen in tissue from DDD patients, leading to the possibility that in addition to local origins, a neuronal origin for TRPV1, most probably from peripheral sensory nerves innervating IVD tissues. As reported previously, TRPV1 is located at the peripheral end of primary sensory nerves and that TRPV1 activates the sensory response by enhancing the release of neurotransmitters or neuromodulators [49,50]. Accordingly, we observed higher CGRP levels in IVD tissues in spite of decreased *CGRP* gene expression, probably in response to TRPV1 activation of CGRP- and SP-positive sensory nerves. Co-expression of CGRP and TRPV1 has been reported in spinal dorsal horn neurons and in DRGs in rat model of lumber disc herniation (LDH), where its association with mechanical allodynia and to spinal neuro-glial activity was observed [51]. 

We observed very strong correlations between *TRPV1* gene expression and *NFKB1* (*r* = 0.823) and *RELA* (*r* = 0.624) mRNA levels in IVD tissues harvested from DDD patients. This association persisted even at the protein level, probably indicating a potential influence of NF-κB signaling on TRPV1 synthesis by IVD cells, or on TRPV1 expression on sensory nerve terminals innervating IVD. Though, TRPV1 expression on sensory nerves can also have an impact on NF-κB signaling in IVD tissues most likely by the release of sensory neuropeptides. Indeed, TRPV1 channels are reported to facilitate the release of CGRP from spinal microglia in rat model of acute inflammatory pain [52]. Moreover, it has been suggested that a Ca^2+^ influx through TRPV1 may modulate IL-6 release and NF-κB nuclear translocation in cultured rat microglia [25]. However, other mechanisms, which may also be Ca^2+^ dependent, can play a significant role in these events as NF-κB activation is highly Ca^2+^ dependent [53]. In addition, *TRPV1* gene expression was positively associated with back pain-related disability in DDD patients, indicating its contribution to nociceptive mechanisms within degenerative discs. The role of TRPV1 in inflammatory pain needs to be further elucidated as the clinical trials with TRPV1 antagonists were not successful due to side effects [54]. However, further studies unfolding the potential role of TRPV1 and its relation to NF-κB signaling in DDD leading to chronic back pain can provide new insight. 

Taken together, our present findings provide evidence of NF-κB activation in DDD. Further, our studies highlight association of NF-κB signaling with SP and TRPV1 expression in IVD tissues, suggesting the role of NF-κB in the pathophysiology of disc degeneration leading to chronic low back pain. 

*Study limitations.* The use of PM human specimens as the control tissue is not ideal, although we did not observe any compromise on the quality of RNA in the PM group assessed as the RQI of PM tissues was not statistically different from those of DDD patients. A similar design has been used previously, demonstrating no effect of PMI on the quality of RNA prepared from trabecular bone [55]. However, the BMI differences between OA and PM controls or the limited number of samples in the PM group could have influenced our results. 

Furthermore, as we did not correct for multiple comparisons, the results regarding correlations between substances as well as with clinical symptoms are to be regarded as exploratory and need to be confirmed in another independent cohort of DDD patients. 

## 4. Materials and Methods 

### 4.1. Study Subjects 

#### 4.1.1. Patients 

Forty patients (22 F/18 M) with DDD at segments L4-S1 (L4-5 from 16 and L5-S1 from 24 patients), undergoing disc replacement or spinal fusion surgery, were included in the study. All patients had radiologically confirmed disc degenerative changes at the treated and examined segments and chronic low back pain (lumbago) as their dominant pain complaint for longer than one year. All patients were recruited from the waiting list for disc replacement or spinal fusion surgeries at the Stockholm Spine Center, Sweden. The exclusion criteria were: chronic pain due to other causes than DDD, such as fibromyalgia, disc herniation, knee or hip osteoarthritis, inflammatory rheumatic or neurological disease, or previous surgery at the investigated segments. 

All participants were informed regarding the study procedure and written consent was obtained. Disc tissues were collected during surgery, AF layers were separated from NP and immediately frozen at −80 °C until analysis. In all biochemical analysis, only AF tissues were used. Twenty patients were taking analgesics, 14 were taking acetaminophen, 3 were on antidepressants, and 2 on anticonvulsants, and 5 had previously been taking non-steroidal anti-inflammatory drugs (NSAIDs), however two weeks prior to the surgical procedure the patients had discontinued the use of NSAIDs. All patients received 1 g acetaminophen (paracetamol) as premedication before surgery and were also given 20 mg oxycodone orally. 

#### 4.1.2. Postmortem Controls 

Eighteen postmortem subjects (6 F/12 M) with no history of disc degenerative disease, disc herniation, osteoarthritis, inflammatory rheumatic or neurological disease were included as controls. Intervertebral discs were excised from the lumbar spine between L4 to S1 (L4-5 from 7 subjects and L5-S1 from 11 subjects). The postmortem interval was on average 49.6 h. Disc tissues were collected, the AF and NP layers were separated and immediately frozen at −80 °C until analysis. The discs were macroscopically examined during the autopsy procedure for any characteristic signs of degeneration. Patient and PM control demographic data are reported in Table 1. 

This study was approved by the Regional Ethical Review Board in Stockholm, Sweden with Reference no. 2011/2036-31-1 (Approval date: 18 January 2012) and Reference no. 2012/2006-32 (Approval date: 14 December 2012) and followed the guidelines of the Declaration of Helsinki.

### 4.2. Questionnaires 

Pain intensity at the back and leg was assessed using a 100 mm visual analogue scale (VAS) where 0 = no pain and 100 = worst pain imaginable. Oswestry Disability Index (ODI) was used to determine the patient’s disability due to low back pain. ODI range from 0 (no disability) to 100 (maximal possible disability) [56]. Clinical parameters were collected 1–2 days prior to surgery while, 9/40 patients had reported data more than 2 weeks before surgery. 

### 4.3. Quantitative Real-Time Polymerase Chain Reaction (qRT-PCR) 

Frozen disc tissues were powdered using a Mikro-dismembrator (B. Braun Biotech International, Melsungen, Germany) in liquid nitrogen, and dissolved in 2–3 volumes of Trizol reagent (Invitrogen Life Technologies Inc., Carlsbad, USA). RNA was then extracted and purified using a RNeasy MiniKit (Qiagen, Sollentuna, Sweden) following the manufacturer’s protocol. Quantification of RNA was determined using a Nanodrop ND-1000 spectrophotometer (Isogen Life Science, Stockholm, Sweden), and RNA quality was measured as the RNA quality index (RQI) using the Experion electrophoresis system (BioRad, Stockholm, Sweden). First-strand cDNA were synthesized from 0.5 μg of total RNA using a first-strand cDNA Synthesis Kit (Roche, Mannheim, Germany). 

RT-qPCR was performed using TaqMan Gene Expression Assays (Applied Biosystems, Carlsbad, CA, USA) with the GeneAmp 7500 Fast Sequence Detection system (Applied Biosystems). Predeveloped specific primers for *RELA* (Hs00153294_m1), *NFKB1* (Hs00765730_m1), *MMP-3* (Hs00968305_m1), *TAC1;* gene which encode for SP (Hs00243225_m1), *CGRP* (Hs01100741_m1), and *TRPV1* (Hs00218912_m1) were used to detect targets. Relative abundance was calculated using a standard curve generated by adding a dilution series of cDNA from TNF-stimulated human fibroblast like synoviocytes (FLS). Data were normalized to the mean of *HPRT* (Hs02800695_m1) and *GAPDH* (Hs00266705-g1) gene expression, and presented as relative expression units. Due to technical or handling complications, detectable RNA was obtained from 35/40 DDD patients and 17/18 PM controls (Table 1). No differences were noted for RNA quality index (RQI) (*p* = 0.541) among patients and PM controls, with values measured as 6.8 ± 0.6 for DDD patients and 6.2 ± 0.7 for PM controls, respectively. 

### 4.4. Cytoplasmic and Nuclear Extraction 

Frozen disc tissues collected from DDD patients and PM controls were powdered using a Mikro-dismembrator in liquid nitrogen, and mixed with hypotonic buffer consisting of 20 mM Tris-HCl, 10 mM NaCl, 3 mM MgCl_2_ containing 10% phosphatase and 10% protease inhibitor cocktail (Thermo Scientific, Stockholm, Sweden). After addition of 10% liquid NP-40, homogenates were centrifuged at 3000× *g* for 10 min at 4 °C. Supernatant fractions corresponding to the cytoplasmic extracts were collected, aliquoted and stored at −80 °C. The pellets were re-suspended in extraction buffer containing 10 mM Tris, 100 mM NaCl, 1 mM EDTA, 1% Triton X-100, and 0.1% SDS deoxycholate supplemented with 2% protease inhibitor cocktail and 1 mM PMSF, incubated at 4 °C for 30 min with vortex mixing every 10 min, followed by centrifugation at 14,000× *g* for 30 min at 4 °C. Supernatants corresponding to nuclear extracts were collected, aliquoted and stored at −80 °C. TNF-stimulated human FLS were included as a positive control for the cytosolic and nuclear protein extraction procedures. Protein content was measured using a BCA Assay Kit (Thermo Scientific). Due to technical and handling complications, cytoplasmic and nuclear extracts could be prepared from disc samples collected from 36/40 patients but 18/18 PM controls (Table 1). Mean protein concentration in cytoplasmic fractions was 3.35 ± 2.47 mg/mL for the patient samples and 2.60 ± 2.25 mg/mL for the PM control samples, while that for the nuclear fractions was 2.49 ± 2.57 mg/mL for the patients and 1.01 ± 0.66 mg/mL for the PM controls with no significant differences between these groups.

### 4.5. Detection of NF-κB1–DNA Binding and RelA Expression by ELISA 

The NF-κB1–DNA binding activity and RelA expression was measured in the nuclear extracts from IVD tissues collected from DDD patients and PM controls using a commercially available ELISA kits (Rockland, Limerick, USA and Invitrogen Life Technologies, Carlsbad, USA). Assays were performed according to the protocol set by the manufacturer. Briefly, the assay employed antibodies specific for NF-κB1 and RelA coated on 96-well plates. All reagents, samples and standards were prepared according to the manufacturer’s instructions. Subsequently, standard or samples were added to each well and incubated at room temperature followed by the addition of a biotin antibody. Next, streptavidin solution was added followed by incubation at room temperature. After addition of the stop solution, absorbance was measured at 450 nm. 

### 4.6. CGRP, SP, and TRPV1 ELISA 

Cytoplasmic extracts from IVD tissues collected from DDD patients and PM controls were used to measure CGRP, SP, and TRPV1 levels. Commercially available ELISA kits for CGRP and SP (Phoenix Europe GmbH or R & D Systems) and TRPV1 (LifeSpan BioScience, Inc., Seattle, USA) were used. Assays were performed according to protocols set by the manufacturer. Briefly, the assay employed antibodies specific for CGRP, SP, and TRPV1 coated on 96-well plates. All reagents, samples and standards were prepared as instructed. Subsequently, 50 μL standard or samples along with 50 μL of biotinylated antibody for CGRP and SP or 100 μL standard or samples for TRPV1 were added to each well (except for the blank controls) and incubated at room temperature, followed by the addition of 50 or 100 μL biotinolyated antibody. Subsequently, a substrate solution was added, followed by incubation at room temperature. After addition of the Stop Solution, absorbance was immediately assessed at 450 nm. 

### 4.7. Statistical Analysis 

The data were analyzed using IBM SPSS Statistics 22 software program International Business Machines Corp., New York, NY, USA). Student’s *t*-test was applied to detect differences between groups. A univariate analysis of covariance was applied to observe age-, gender-, or BMI-related group differences. The significance of correlations was determined by Spearman correlation co-efficient and effects of age, gender, and BMI were determined by partial correlation analysis. A *p*-value ≤ 0.05 was considered significant. 

## Figures and Tables

**Table 1:** Demographic and clinical characteristics of degenerative disc disease (DDD) patients and postmortem (PM) controls included in study. Data presented as mean ± Standard Deviation. (BMI = body mass index; Pain VAS = visual analogue scale; ODI = Oswestry Disability Index; n.s = non-significance; - = not applicable; qRT-PCR = quantitative real-time polymerase chain reaction; ELISA = enzyme-linked immunosorbent assay) Pain VAS = 0–100, worst = 100; ODI = 0–100, worst = 100.

Subject Characteristics		DDD Patients	PM Controls	Differences
Study subjects (N)		40	18	
Average Age (years ± SD)		44.5 (± 10.0)	42.7 (± 13.0)	n.s
Gender (F/M)		22/18	6/12	
BMI (kg/m^2^)		25.4 (± 5.4)	28.37 (± 5.1)	*p* = 0.0001
Post-mortem interval (h)		-	49.6 (± 15.5)	
Anti-nociceptive medication		20/40		
VAS Back (mm)		32.01 (± 19.98)	-	
VAS Leg (mm)		4.71 (± 9.37)	-	
Oswestry Disability Index (ODI)		31.59 (± 12.25)	-	
Subjects included in biochemical analysis				
qRT-PCR	N	35/40	17/18	
	Gender (F/M)	18/17	6/11	
	Age years (±SD)	45.17 (±9.7)	41.7 (±12.8)	n.s
ELISA	N	36/40	18/18	
	Gender (F/M)	19/17	6/12	
	Age years (±SD)	44.30 (±9.2)	42.7 (±13.0)	n.s
